# Comparative transcriptome analysis of cells from different areas reveals ROS responsive mechanism at sclerotial initiation stage in *Morchella importuna*

**DOI:** 10.1038/s41598-021-87784-w

**Published:** 2021-05-03

**Authors:** Qizheng Liu, Guoqiang He, Jinkang Wei, Caihong Dong

**Affiliations:** 1 State Key Laboratory of Mycology, Institute of Microbiology, Chinese Academy of Sciences, NO.3 1st Beichen West Road, Chaoyang District, Beijing, 100101 China; 2 Beijing Agricultural Technology Extension Station, Beijing, 100029 China

**Keywords:** Applied microbiology, Fungal biology

## Abstract

Morels are some of the most highly prized edible and medicinal mushrooms, with great economic and scientific value. Outdoor cultivation has been achieved and expanded on a large scale in China in recent years. Sclerotial formation is one of the most important phases during the morel life cycle, and previous reports indicated that reactive oxygen species (ROS) play an important role. However, ROS response mechanisms at sclerotial initiation (SI) stage are poorly understood. In this study, comparative transcriptome analyses were performed with sclerotial and hyphal cells at different areas in the same plate at SI stage. Gene expression was significantly different at SI stage between sclerotial formation and mycelia growth areas. GO and KEGG analyses indicated more vigorous metabolic characteristics in the hyphae area, while transcription process, DNA repair, and protein processing were enriched in sclerotial cells. Gene expression related to H_2_O_2_ production was high in the hyphae area, while expression of H_2_O_2_-scavenging genes was high in sclerotial cells, leading to a higher H_2_O_2_ concentration in the hyphal region than in the sclerotium. Minor differences were observed in gene expression of H_2_O_2_-induced signaling pathway in sclerotial and hyphal cells; however, expression levels of the target genes of transcription factor MSN2, important in the H_2_O_2_-induced signaling pathways, were significantly different. MSN2 enhanced stress response regulation in sclerotia by regulating these target genes. Small molecular HSPs were also found upregulated in sclerotial cells. This study indicated that sclerotial cells are more resistant to ROS stress than hyphal cells through transcriptional regulation of related genes.

## Introduction

Morels (*Morchella*, *Ascomycota*), some commercially important edible mushrooms, are sclerotium-forming fungi. Morel cultivation has been a research focus worldwide for more than 100 years, and outdoor cultivation of *Morchella* belonging to the Elata Clade, including *M. importuna*, has succeeded and expanded rapidly in recent years in China^1^.

Sclerotial formation is an important phase during the morel life cycle^2^, and growers judge spawn quality based on sclerotium quantity empirically in field cultivation^1^. The success of morel cultivation in China is mainly attributed to two factors: cultivable strains prone to fructify^3^ and the implementation of exogenous nutrient “feeding technology”^4,5^. “Feeding technology” in morel field cultivation is based on extensive cultivation experience and the principle of sclerotia formation^4^. However, sclerotial formation mechanisms in *M. importuna* remain elusive.

Sclerotia are compact masses of fungal mycelia that serve as survival structures for ascomycetes and basidiomycetes^6^. Many fungi produce sclerotia during their life cycle, with 85 fungal genera in at least 20 orders of *Basidiomycota* and *Ascomycota* reported to be sclerotium-forming fungi^6^. Typically, there are three stages during sclerotial formation: sclerotial initiation (SI), sclerotial development (SD), and sclerotial maturation (SM)^7^. Sclerotial metamorphosis is believed to be induced by oxidative stress; therefore, the hypothesis has been put forward that reactive oxygen species (ROS) induce sclerotial formation^7^.

Sclerotia of morels were formed by repeated hyphal branching and further expansion and coalescing to form a single large sclerotium. They are described as pseudosclerotia^2^ as they do not have structures typical of sclerotia, such as those produced by *Sclerofinia sclerotiorum*, characterized by the medulla and rind. ROS outbreak can promote sclerotial formation of morels^8^. Previous studies found that a hydrogen peroxide (H_2_O_2_) concentration of 20 mM could promote sclerotial initiation by H_2_O_2_- induced MAPK signaling pathway activation^9^. Recently, transcriptome analysis of samples from three developmental stages of sclerotial formation (vegetative mycelia, initial sclerotia, and mature sclerotia) in *M. importuna* showed that differentially expressed genes were mainly involved in primary metabolism^4^.

Our previous study found that distinct boundaries appeared between mycelia and sclerotia areas in the SI phase when *M. importuna* was incubated on PDA plates. Cell morphologies and ROS stresses differed between the two areas^9^. However, the mechanism by which ROS control morphogenesis is unclear. In this study, comparative transcriptomic analysis was performed with samples from two parts with different cell morphologies at the SI stage of *M. importuna*. In contrast to transcriptome analysis of different sclerotial developmental stages^4^, gene expression in cells from different areas (i.e., sclerotial and hyphal cells) at the same SI stage were compared, highlighting the characteristics of gene expression in sclerotial cells. Our aim was to elucidate key metabolic pathways and related genes regulating the formation of sclerotium and explain the mechanisms by which ROS regulate sclerotial formation.

## Results

### Morphology comparison between inside and outside of plates at SI stage

**Figure 1:**
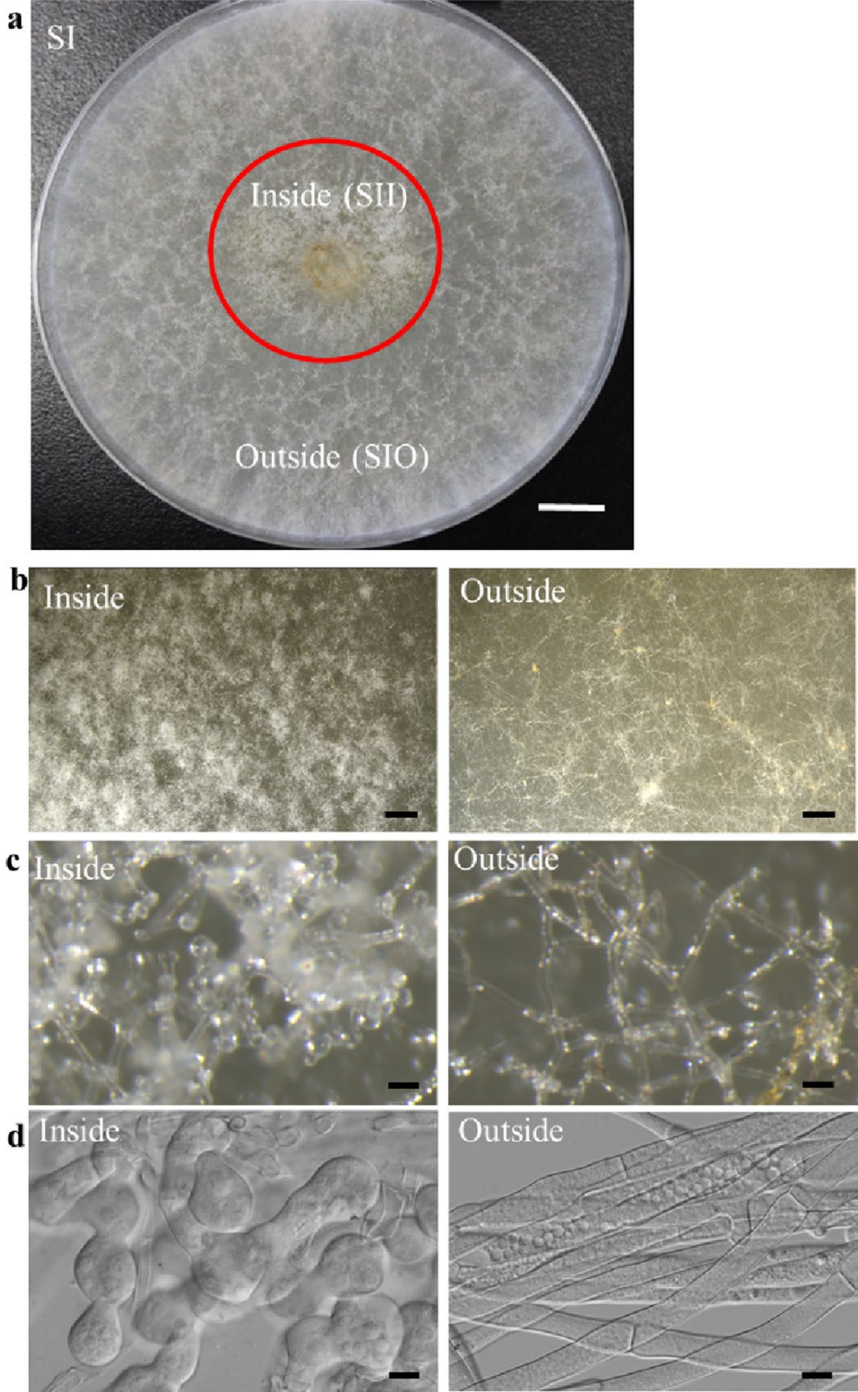
Morphological observation of SII and SIO cells cultured on PDA medium. (**a**) Colony morphology of *M. importuna* cells grown on PDA plates at SI stage. An area of 2 cm radius around the center which was circled with red was divided as the inside, and the remaining of the plate was divided as the outside area. There was distinct boundary. Size bar = 1 cm. (**b**) Magnified pictures of colonies of SII and SIO. Size bars = 1 mm. (**c**) Sclerotium observation in SII and SIO under the stereoscope. Size bars = 30 µm. (**d**) Cell morphology of *M. importuna* in SII and SIO at SI stage. Size bars = 10 µm.

According to previous observation, *M. importuna* growth and sclerotial formation can be divided into five distinctive phases: hypha early (HE), hyphal growth (HG), SI, SD, and SM^9^. After *M. importuna* strain was cultured on a 90-mm diameter Petri dish with PDA medium at 20 °C for 4 days, colonies entered the SI phase. There was a significant difference in cell morphology between the inside and outside of plates and distinct boundaries (Fig. 1a). Mycelia inside plates at the SI (SII) were dense (Fig. 1b), cells expanded as nearly spherical and bead-like, and there was obvious micro-sclerotia when observed under the stereoscope and microscope (Fig. 1c,d). It should be sclerotial growth area. Cell expansion in SII is a unique morphological characteristic during sclerotial formation as these cells expand and proliferate, rapidly increasing the sclerotia biomass, into the SD stage. Outside mycelia (SIO) were sparse, seen as normal mycelia (Fig. 1b), and there was no obvious micro-sclerotium (Fig. 1c,d). It was shown as a hyphal growth area. Mycelia of SII and SIO (Fig. 1a) from two Petri dishes were collected and mixed, respectively, for transcriptome sequencing.

### Gene expression profiles of inside and outside cells at SI phase of *M. importuna*

To compare SII and SIO gene expression profiles, transcriptome sequencing was conducted using samples from different areas with two biological repeats. Four cDNA libraries were prepared and subjected to Illumina deep sequencing, and the results of Pearson correlation analysis of sequencing data (Supplemental Fig. S1) indicated good repeatability. Illumina paired-end sequencing generated 125.2 million raw read pairs. After cleaning and quality checks, 120.8 million clean read pairs were obtained. All Q30 percentages for sequences (with an error probability of 0.001, a high-quality indicator) in the four libraries were over 94 (Supplemental Table S2). Over 94% of reads for each sample could be mapped to the *M. importuna* genome (Supplemental Table S3).

Using FPKM (Fragments Per Kilobase of Exon Per Million Fragments Mapped) cutoff values of 1, 74.9% and 74.3% genes were expressed in the SII and SIO samples, respectively. On a global scale, all genes could be divided into four categories according to their FPKM values, with the majority of genes moderately expressed (10 ≤ FPKM < 100) in all samples. Genome-wide distribution of gene transcription levels was similar in both groups (Supplemental Table S4).

### Identification of differentially expressed genes between samples from different areas

**Figure 2:**
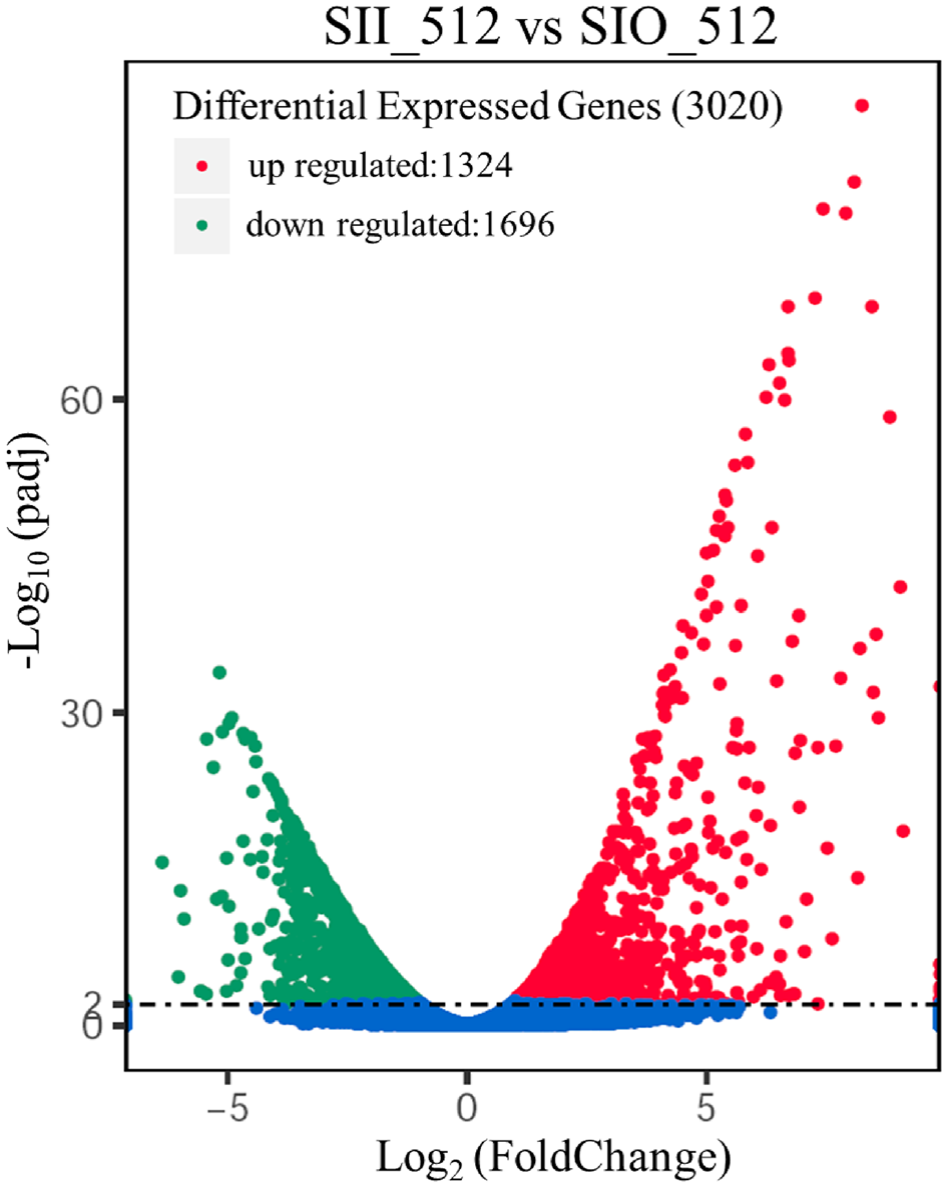
Volcano of differentially expressed genes between samples from SII and SIO.

A total of 3020 genes were significantly differentially expressed between two areas (SII vs. SIO), representing 26% of *M. importuna* genes (Supplemental Table S5). A total of 1324 genes were upregulated and 1696 genes downregulated when SII was compared with SIO (Fig. 2). Where the FPKM value of a gene in SII or SIO was greater than 1 and the other was less than 1, the gene was considered to be a uniquely expressed gene. There were 187 and 147 uniquely expressed genes in SII (1.61% of the genome) and SIO sample (1.27% of the genome), respectively, with most genes expressed at low levels (Supplemental Fig. S2 and Supplemental Table S6). Only three genes (JGI 492735, JGI 568718, and JGI 545084) expressed uniquely in SII belonged to the high expression level group (FPKM > 100). The gene JGI 492735 encodes gamma-glutamyl putrescine oxidoreductase, and the other genes encoded hypothetical proteins.

### GO and KEGG analysis of DEGs

**Figure 3:**
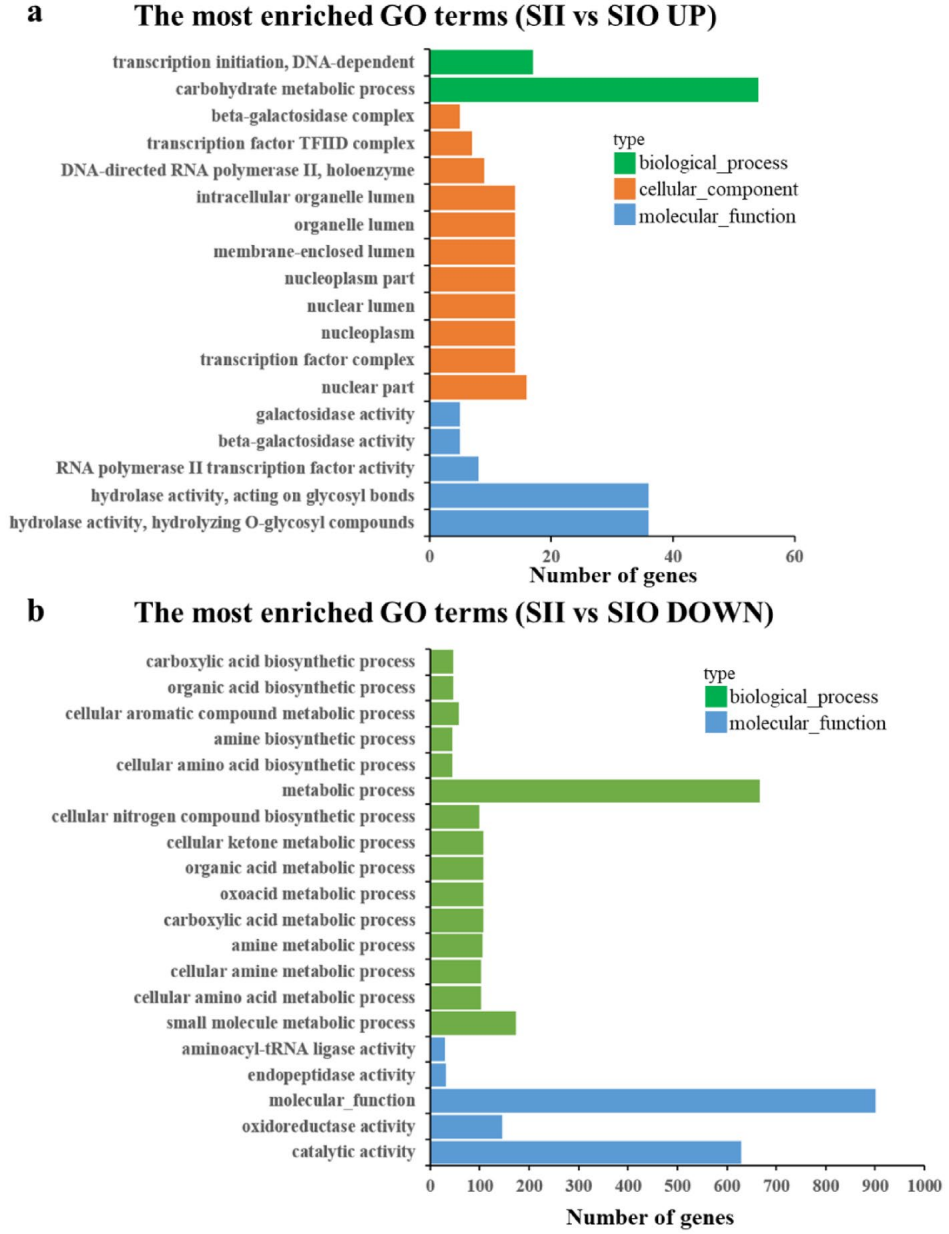
The most enriched GO terms of up and down DEGs compared between SII and SIO. All 18 in up and the top 20 (ranked with *P* value) GO terms in down were shown.

Eighteen and 114 GO terms were enriched for up- and downregulated genes, respectively, when compared between SII and SIO (*P* < 0.01, Supplemental Table S7). In the upregulated group (Fig. 3), enriched terms were mainly related to transcription processes, including RNA polymerase II transcription factor activity (GO: 0003702), transcription factor complex (GO: 0005667 and 0005669), DNA-directed RNA polymerase II (GO: 0016591), and transcription initiation (GO: 0006352). Others were related to carbohydrate metabolism, including hydrolase activity on glycosyl bonds (GO: 0004553 and 0016798), galactosidase activity (GO: 0004565, 0015925, and 0009341), and carbohydrate metabolic process (GO: 0005975). Organelles and nuclear lumen (GO: 0031981, 0043233, and others) were also enriched. In the downregulated group, most enriched terms were involved with metabolic (GO: 0044281, 0006520 and 0044106), biosynthetic (GO: 0008652, 0009309, and 0016053), and catalytic activity (GO: 0003824). Some terms related to oxidoreductase activity, including GO: 0016491, 0016614, 0016616, and 0015036.

DEGs were mapped to the KEGG database and tested for enrichment. Ten and 13 pathways were enriched in up- and downregulated genes when compared between SII and SIO, respectively (*P* < 0.05, Supplemental Fig. S3 and Supplemental Table S8). The enriched pathway of upregulated genes included protein processing in the endoplasmic reticulum (tml04141), N-glycan biosynthesis (tml00510), and starch and sucrose metabolism (tml00500). The top three enriched pathways in downregulated genes were involved in biosynthesis of secondary metabolites (tml01110), biosynthesis of amino acids (tml01230), and glycolysis/gluconeogenesis (tml00010).

### Expression of carbohydrate active enzyme genes

**Figure 4:**
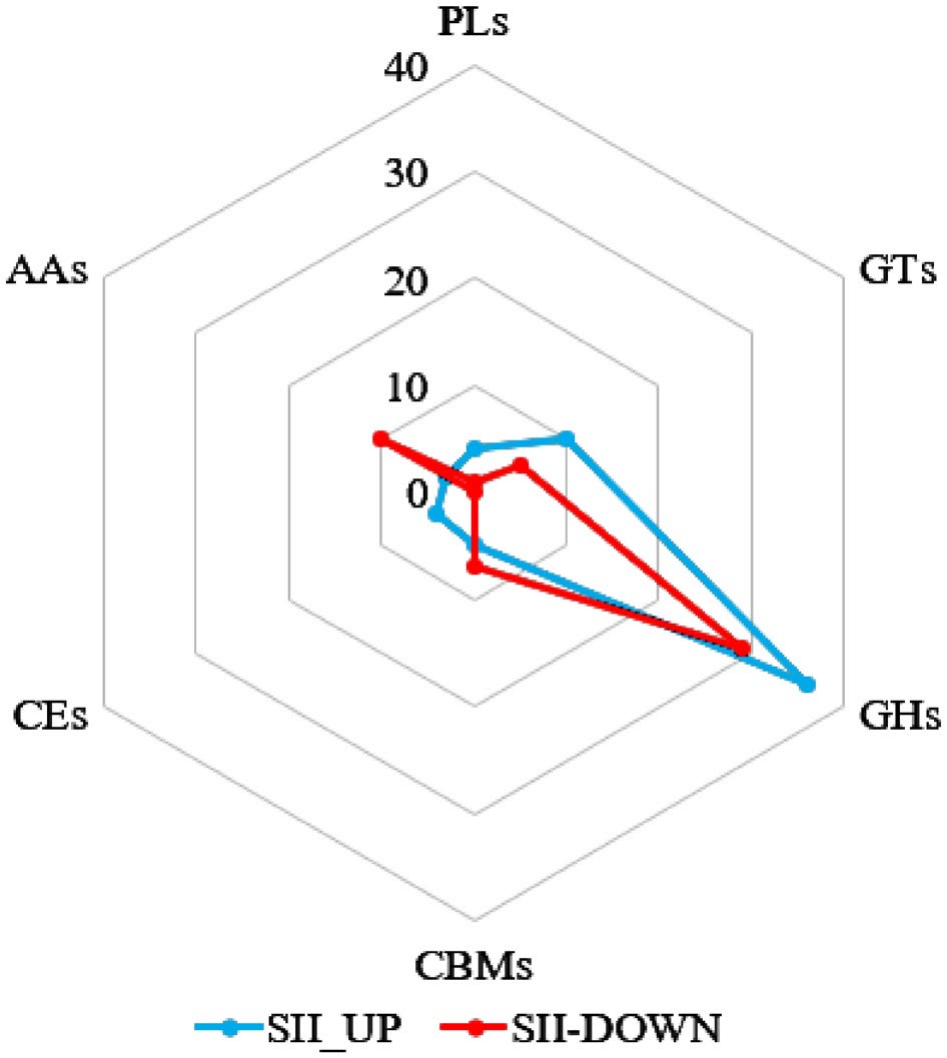
Radar chart showed DEGs numbers of each classes in CAZy familys. Total DEGs statistics in each CAZy family are shown with a radar chart. The dot in the radar axis represents the number of DEGs, and detailed information is shown in Table S9. Blue and red dots indicate UP and DOWN group in the SII sample, respectively. AA: Auxiliary Activity Family, CEs: Carbohydrate Esterase Family, CBMs: Carbohydrate-Binding Module Family, GHs: Glycoside Hydrolase Family, GTs: Glycosyl Transferase Family, PLs: Polysaccharide Lyase Family.

Among 350 carbohydrate active enzyme (CAZy) genes^10^, 114 were differentially expressed, accounting for 32.6% (Supplemental Table S9), among which 62 were upregulated. The highest number of CAZy genes was in the glycoside hydrolase family (GHs, 161, 65 of which are DEGs), the second highest number was in the glycosyl transferase family (GTs, 68, 15 DEGs), the third was in the auxiliary activity family (AAs, 51, 13 DEGs), followed by the carbohydrate binding module family (CBMs, 34, 12 DEGs), polysaccharide lyase family (PLs, 20, 5 DEGs), and carbohydrate esterase family (CEs, 16, 4 DEGs) (Fig. 4 and Supplemental Table S9). Among genes upregulated in SII, one CBM family glycoside hydrolase gene (JGI 571377) had an FPKM value greater than 1000. Among the downregulated genes for SII, ricin B-like lectins of the CBM family (JGI 534286), pyranose oxidase (JGI 512223) of the AAs family, alpha-1, 4-glucan lyase (JGI 604718) of the GHs family, and glycosyl transferase (JGI 488814) of the GTs family had FPKM values greater than 1000 (Supplemental Table S9). The sum of FPKM values of JGI 534286 and JGI 512223 accounted for more than half of the total FPKM value in SIO (Supplemental Fig. S4), indicating that these two genes play an important role in mycelial cells.

### Expression analysis of genes encoding antioxidant enzymes

**Figure 5:**
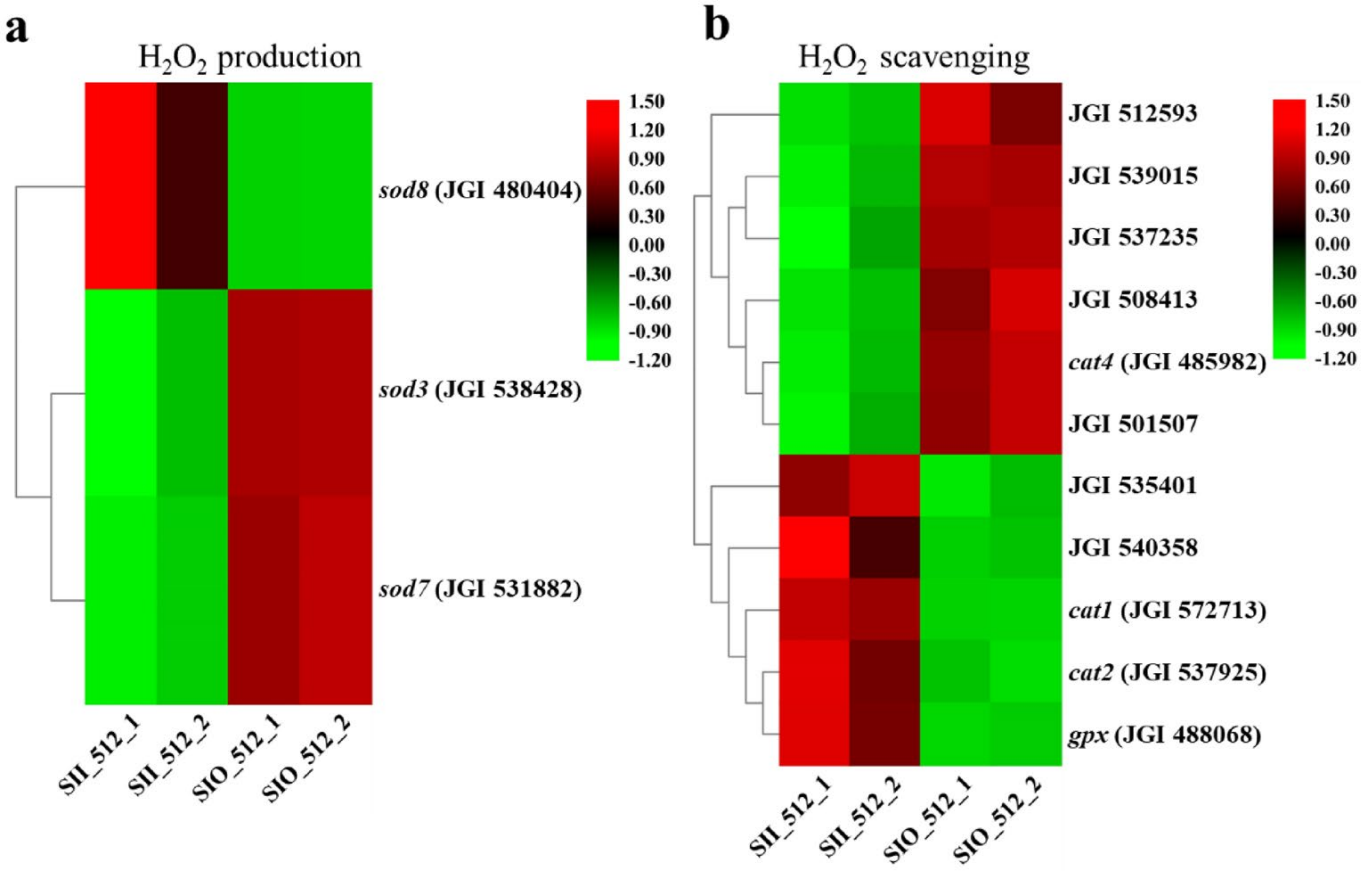
Expression level of DEGs related to H_2_O_2_ metabolism. (**a**) Expression of DEGs related to H_2_O_2_-production. (**b**) Expression of DEGs related to H_2_O_2_-scavenging.

Sclerotial metamorphosis is believed to be induced by oxidative stress^11^. When cells are subjected to oxidative stress, antioxidant systems directly eliminate ROS. Superoxide dismutase (SOD) can eliminate superoxide anions (O_2_^−^) and produce H_2_O_2_, which is then eliminated by catalase, peroxiredoxin, and glutathione peroxidase (GPX). All 28 genes related to H_2_O_2_ metabolism are expressed in both SII and SIO, and half of them were DEGs (Supplemental Table S10). Among DEGs related to H_2_O_2_ production (Fig. 5), *sod3* and *sod7* were downregulated, but *sod8* was upregulated in SII. Meanwhile, only the *sod3* gene was highly expressed. Catalase, thioredoxin, and peroxiredoxin are enzymes capable of directly reducing peroxides^12^. There were 11 DEGs (Supplemental Table S10), with 5 and 6 genes up- and downregulated, respectively (Fig. 5 and Table S10). Mitochondrial peroxiredoxin *prx1* (JGI 508413) showed the highest downregulation in SII was compared with SIO (fold of − 4.05 and Q value of 9.8046E^−24^). As showed in Fig. S5, total FPKM value of *sod* DEGs in SIO was higher than in SII. However, *cat* and other DEGs encoding enzymes for H_2_O_2_ elimination have a much higher total FPKM value in SII than that of SIO.

Trehalose has been reported to protect cells against oxygen radicals^13^. The gene encoding for putative trehalose synthase (JGI 478274) was upregulated by 5.85-fold (Q value of 1.24E^−54^) in SII compared with SIO (Supplemental Table S11). Vitamin B6 prevents oxygen radical generation and lipid peroxidation caused by H_2_O_2_ in cells^14^, and vitamin B6 biosynthesis gene (JGI 481444) were downregulated (− 2.77 and 3.44E^−09^) (Supplemental Table S11).

### Differential expression of genes encoding heat shock proteins

**Figure 6:**
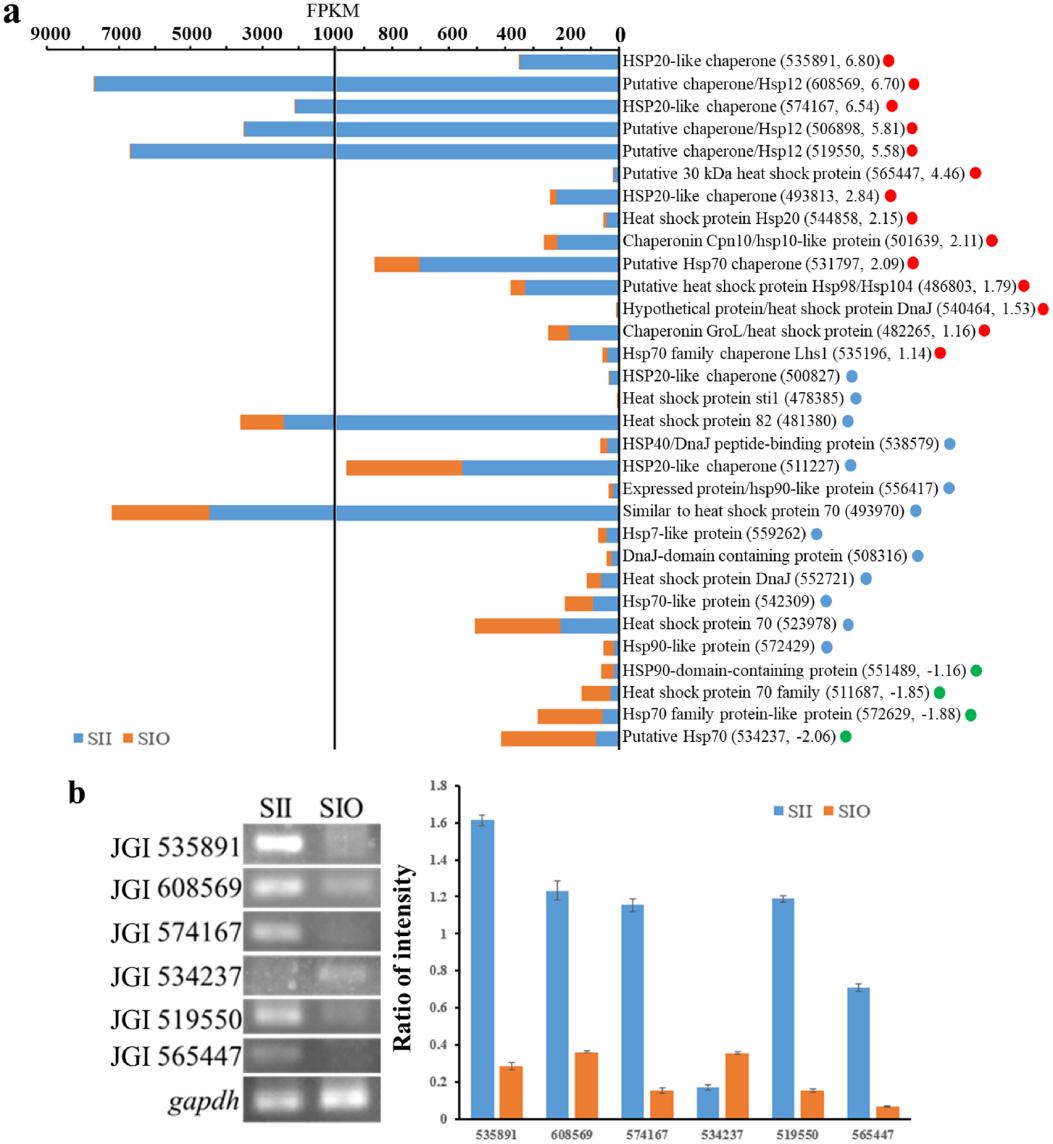
Expression levels of *hsp* genes. (**a**) FPKM of *hsp* genes in SII and SIO samples. Blue and orange bars indicate FPKM of genes in SII and SIO, respectively. Up-regulated genes were labeled as red dots followed by gene name, blue dots were non-DEGs, and down-regulated genes were labeled as green dots. The number in the bracket indicates the gene number of JGI log2 (Fold Change) value. (**b**) Expression of several *hsp* genes verified by RT-PCR. Constitutive *gapdh* served as the control. Intensity ratio indicated *hsp* gene expression versus *gapdh* expression. Error bars indicate standard deviation.

Molecular chaperones (heat shock proteins, HSP) can make the protein fold correctly, prevent protein denaturation, and restore its original spatial conformation and biological activity^15,16^. All 31 HSP genes in the reference genome^10^ were detected in samples, 18 of which showed differential expression. Except for two members of the hsp70 family, all genes upregulated in SII were small HSPs (marked with red dots in Fig. 6a). JGI 535891, 608569, and 574167 showed the highest fold change (more than 6) (Supplemental Table S12). Genes encoding HSP with large molecular weights (marked with green dots in Fig. 6a) were downregulated in SII, and JGI 534237 showed the top1 fold change (−2.06 and 3.16E^−08^). Expression of several *hsp* genes was verified by RT-PCR (Fig. 6b and Table S12), and the original figures are shown in Fig. S7.

### Gene expression related to H_2_O_2_-induced signal pathway

In response to oxidative stress, relevant signaling pathways and transcription factors begin to be activated, further enhancing the antioxidant capacity of cells^17,18^. It was reported that MAPK and cAMP pathways are the main signaling pathways of H_2_O_2_ response in filamentous fungi^11^, and the expression of related genes was analyzed. Among cell membrane receptor genes encoding histidine kinases (*mak1*, *sln1*) and G protein-coupled receptors (*gpcr*), only the transcription level of *gpcr* was significantly different (Supplemental Table S13). There were differences in transcription levels of MAPK pathway-related genes (*fus3*, *mcs4*), but no difference was observed for most of them (*hog1*, *spm1*, *mpr*, *cdc25*, *pka-c1*, *pka-c2*, *pka-r*, *ac*) (Supplemental Table S13). The activated signaling pathway regulates transcription factors; however, there was no differential expression between SII and SIO among the potential downstream transcription factors, including *msn2*, *yap1*, *prr1*, *pap1*, and *atf1* (Supplemental Table S13). Expression levels of some genes were verified by qRT-PCR, as shown in Fig. S6a.

**Figure 7:**
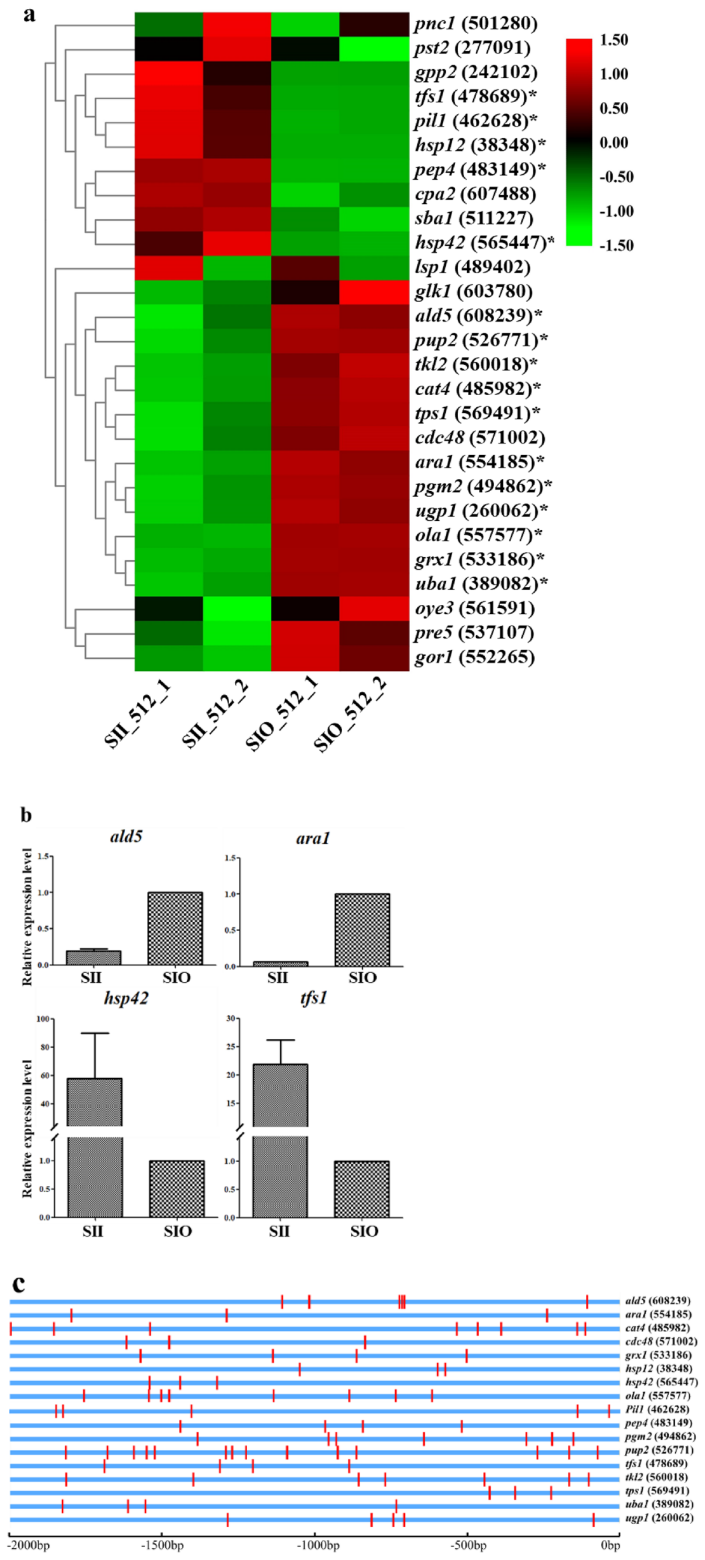
Analysis of transcriptional factor MSN2 target genes. (**a**) Heatmap of MSN2 target gene expression levels. Asterisk (*) indicates DEGs. The number in the bracket indicates the gene number of JGI. (**b**) Relative expression levels of some DEGs of MSN2 target genes revealed by qRT-PCR. (**c**) Position of STRE in promoter of DEGs in MSN2 target genes. Vertical red bands indicated STRE.

It has been reported that transcription factor MSN2 can activate genes in response to several environmental and metabolic cues, including H_2_O_2_, heat, osmotic, and acidic stresses^19,20^ in *Saccharomyces cerevisiae*. Using BLASTP in the *M. importuna* genome with the sequences of target genes of MSN2 in *S. cerevisiae*^21^, homologous proteins were obtained and the expression levels of target genes were analyzed. Compared with that in SIO, expression levels of more than half of the target genes of MSN2 were significantly differentially expressed (Fig. 7a and Supplemental Table S14) in SII. Expression levels of some genes (*ald5*, *ara1*, *hsp42*, *tfs1*) revealed by qRT-PCR, as shown in Fig. 7b, confirmed transcriptome analysis results. Promoter sequences up to 2.0 kb upstream from the translation start site of each target gene of MSN2 were scanned using PlantCare program (http://bioinformatics.psb.ugent.be/webtools/plantcare/html/) for the identification of cis-acting regulatory elements. Over ten kinds of cis-elements for each target gene and CAAT-box, STRE (stress response promoter element, AGGGG or CCCCT), and Unnamed_4 (CTCC) motifs were present in the promoters of all target genes (Table S15). STRE cis-element distribution within 2 kb upstream of the DEGs among the MSN2 target genes is shown in Fig. 7c and 3–15 STRE copies are presented. Generally, DEGs of MSN2 target genes were mainly involved in metabolism, response to DNA replication, and oxidative stress. The FPKM value of most DEGs in SII or SIO was more than 100, which were highly expressed genes. Four of these DEGs have FPKM values greater than 1000, including *ald5* (mitochondrial aldehyde dehydrogenase), involved in the regulation or biosynthesis of electron transport chain components and acetate formation^22^, and *ugp1* (UDP-glucose pyrophosphorylase), involved in a wide variety of metabolic pathways and oxidative stress resistance^23,24^, *pep4* (vacuolar aspartyl protease), required for post-translational precursor maturation of vacuolar proteinases and important for protein turnover after oxidative damage^25^, and *hsp12*, involved in maintaining organization during stress conditions^26^.

## Discussion

The imbalance of ROS metabolism leads to high stress and different cellular responses, including signaling, detoxification, cell cycle arrest, and apoptosis^27^. ROS play an important regulatory role in the formation of fungal sclerotia^7^. A significant difference in the morphology and levels of ROS between the cells of SII and SIO was first reported in *M. importuna*^9^. In this study, sclerotia response to ROS and sclerotial formation regulating mechanisms by H_2_O_2_-induced pathway at a genome-wide scale at the SI stage of *M. importuna* were analyzed. Sclerotial cells showed stronger ability for stress resistance, whereas mycelial cells showed vigorous primary metabolism. Transcription factor MSN2 in H_2_O_2_-induced pathway showed an enhanced regulating ability on stress response in SII. This data indicated that ROS resistance in cells was closely related to sclerotial initiation.

### Stress resistance ability in sclerotial cells was stronger

More than 3, 000 DEGs were identified, indicating that different morphological differentiation between hyphal and sclerotial cells at the same SI stage was accompanied by the differential gene expression. The top 20 enriched GO terms of down-regulated genes (SII vs SIO) were mainly related to primary metabolism and catalytic activity. It was reported that turgor which drove mass flow^28^ in mycelia was higher than in sclerotial at SI stage, consistent with vigorous metabolic characteristics in the hyphae area from GO and KEGG analyses, including carboxylic acid metabolic process (GO: 0019752), metabolic process (GO: 0008152), biosynthesis of secondary metabolites (tml01110) and fructose and mannose metabolism (tml00051).

Enriched GO terms of up-regulated genes were mainly related to the transcription process, fundamental biochemical processes, and carbohydrate metabolism. KEGG pathway analysis showed that DNA replication (tml03030), base excision repair (tml03410), N-glycan biosynthesis (tml00510), and various types of N-glycan biosynthesis (tml00513) were in the upregulated group. The base excision repair pathway maintains genome integrity by repairing damaged DNA bases^29^. It was reported that N-glycan related pathway played an important role in quality control of protein folding^30^. These characteristics indicated that sclerotial cells showed a stronger ability to maintain intracellular stability when cells faced external stress which may lead to be out of control of DNA and proteins process.

### Characteristics of differential gene expression of SI stage cells

Different CAZy family genes play important roles in the vegetative growth or fruiting formation of *M. importuna*^4,31^. Results indicated different expression levels of the CAZy family in mycelia and sclerotium at the SI stage, among which the highest number of DEGs belonged to the GHs family. The up-regulation of GHs family members in sclerotial cells may contribute to glycoside and other substance degradation^32^, and be conducive to rapid cell expansion by changing cell wall structure during sclerotium formation. There were four DEGs of UDP-glycosyltransferase in the GTs family, and three of them (JGI 564595, 497672 and 534057) were upregulated in sclerotium. UDP-glycosyltransferase gene expression was correlated with mitosis and was strongly induced in dividing cells in pea and alfalfa^33^. It has also been reported that UDP-glycosyltransferase expression decreased under hydrogen peroxide stress in *Populus tomentosa*^34^. Additionally, some genes related to antioxidants were highly expressed in the mycelium region, including vitamin B6 biosynthesis gene (JGI 481444), while others were highly expressed in the sclerotia region, including putative trehalose synthase encoding gene (JGI 478274). This indicated that there were differences in mycelial and sclerotial cell response to ROS stress.

### Many cells of SII were in G2 phase

Approximately 3% of genes were uniquely expressed in sclerotial or hyphal cells, but most genes had very low expression levels with unknown function. It is worth highlighting that G2-specific kinase (JGI 604040) is one of the uniquely expressed genes in SII, indicating that many cells of sclerotium at the SI stage are in G2 phase. The G2 phase is the anaphase of DNA synthesis, which is the preparatory phase of mitosis. During this period, DNA synthesis was stopped and large amounts of RNA and proteins were synthesized. In the SD period after the SI stage, sclerotial cell numbers increased greatly, requiring many cell proliferation and material preparation. This was consistent with the GO analysis of the DEGs. In the SII samples, many RNA-related GO terms were annotated by highly expressed genes. G1/S-specific cyclin *pcl5* (JGI 42090) was upregulated in hyphae compared with sclerotia, indicating that cells in the hyphae area were in the G1/S phase. Results matched the characteristics of hyphal cells in which metabolism was vigorous. There are subjective differences in morphological observations among different researchers. Therefore, in-depth study of uniquely genes and their expression levels at different stages can be considered to serve as the gold standard for the classification of sclerotia development stage.

### sHsps play an important role in sclerotial formation

Analysis of HSPs revealed that 9 of the 14 upregulated genes were small HSPs^35^ (sHSPs). The top1 of log_2_ (Fold Change) value was JGI535891 (fold of 6.80, Q value of 1.08E^−3^), one of the Hsp20 family members reported to be involved in ROS detoxification in *Methanospirillum hungatei*^36^, and the reduction of ROS accumulation in mice^37^. Second, JGI 608569 (fold of 6.70, Q value of 1.3015E^−69^) from the Hsp12 family was found, which played a role in maintaining plasma membrane integrity during oxidative stress^38^. Hsp10 (JGI 501639) was in up-regulated group, and reported to stabilize catalytic subunit of DNA polymerase-α^39^. Hsp104, that played a role in external stress tolerance^40^, was also up-regulated. Members of the Hsp70 family reported to play a role in initial folding of nascent polypeptide and ATPase activity^41^, showed both up and down-regulation. Only one Hsp90 was found in down-regulated genes, and it controlled yeast to mycelium dimorphism in *Paracoccidioides brasiliensis*^42^. Hsp70-Hsp90 chaperone cascade preference in protein folding was reported in eukaryotic cells^43^. Only four macromolecules *hsp* genes were down regulated in SII vs SIO, i.e. these genes were up-regulated in mycelial cells. These macromolecules *hsp* genes may play a role in maintaining mycelium cell stability under high ROS stress, but can’t promote sclerotial development. It can be concluded that sHSPs may play an important role in sclerotia formation.

### Expression levels of the H_2_O_2_ metabolism genes were consistent with H_2_O_2_ concentration distribution

Previous studies have shown that H_2_O_2_ plays an important role in regulating sclerotial formation and accumulates in *M. importuna* hyphal growth area^9^. Expression of H_2_O_2_ metabolic gene was analyzed^12,44^. Total gene expression of H_2_O_2_ production was high in mycelia, while gene expression of H_2_O_2_ scavenging was high in sclerotia (Supplemental Fig. S5). This trend would lead to a higher H_2_O_2_ concentration in the hyphae region than the sclerotium, consistent with previous reports^9^. From expression levels analysis, among the H_2_O_2_ metabolism related genes, *sod3* (JGI 538428) may play an important role in H_2_O_2_ production by mycelia, while *cat2* (JGI 537925) may be important in H_2_O_2_ scavenging by sclerotia, suggesting these two genes have a key impact at the SI stage.

### MSN2 showed enhanced regulating ability on stress response in sclerotia

Expression levels of genes related to the H_2_O_2_-induced signaling pathway were analyzed, and few differences were found between the hyphal and sclerotial cells. For genes with different expression levels, fold change was small, *gpcr* (− 1.03), *fus3* (− 1.49), and *mcs4* (− 1.37) (Supplemental Table S13). However, expression levels of MSN2 target genes were significantly different in mycelial and sclerotial regions. More than ten different cis-elements for each target gene revealed functional multiplicity of MSN2 target genes, providing insight into gene regulation. It was reported that MSN2 is a STRE-binding protein that activates STRE-regulated genes in response to stress in *Saccharomyces cerevisiae*^20^. In the present study, STRE appeared for 3–15 copies in the promoters of all MSN2 target genes in *M. importuna*, indicating that the function of MSN2 regulating gene expression in response to stress was conserved in fungi. Some target genes were highly expressed in the sclerotia, while others in mycelia. The absolute fold change value ranged from 1 to 2 in mycelia, but main up-regulation in sclerotia was between 3 and 5. High FPKM values were observed in up-regulated genes (Supplemental Fig. S6b), including as *pep4* and *hsp12*. These target genes have shown a role in stress resistance in previous reports. *pep4* is important for protein turnover after oxidative damage^25^ and can reduce accumulation of ROS in *S. cerevisiae*^45^. *tfs1* is an inhibitor of Ras GAP (Ira2p) and lipid-binding domain containing protein, and high-level expression was shown in wide type cells when faced with heat shock and hydrogen peroxide stress in *S. cerevisiae*^46^, reported to contribute to cell membrane stability^47^. *hsp42* is a player for protein homeostasis under physiological and stress conditions^48^. *hsp12* has been reported to be important in maintaining cell membrane integrity^26^ and has been found to be associated with trehalose accumulation in *S. cerevisiae*^49^. All these up-regulated genes showed anti-stress function, indicating that MSN2 enhanced stress response regulation in sclerotia by regulating these target genes. Additionally, it was suspected that the transcription factor MSN2 regulated target gene expression levels mainly through posttranslational modifications, such as phosphorylation^50^ or other factors, not only the amount of protein, which was our ongoing research.

### ROS regulated the morphogenesis of sclerotia

**Figure 8:**
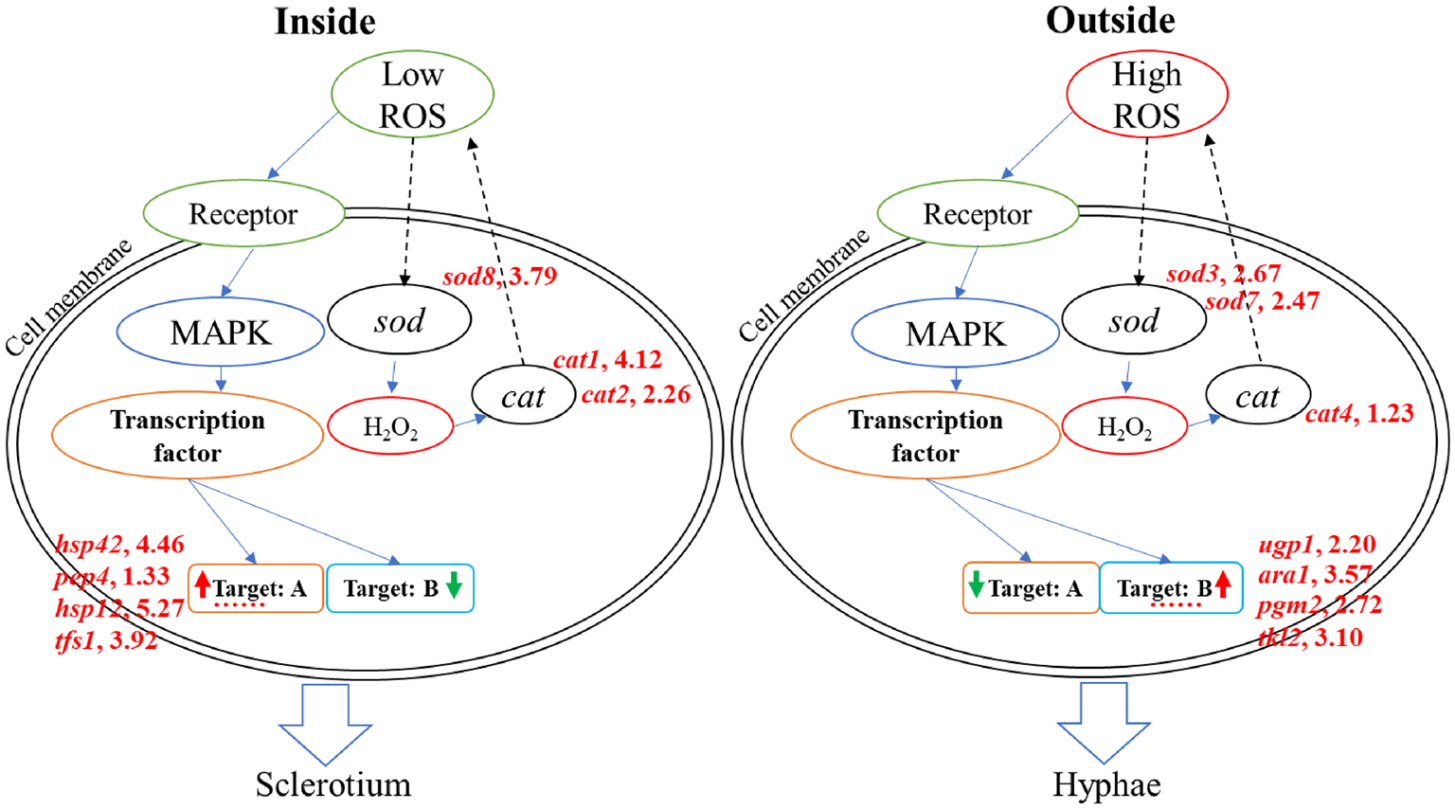
Model of morphogenesis caused by different ROS stress at sclerotial initiation stage in *M. importuna*. The receptor in the cell membranes receives signal and transmits to kinases that control the expression of transcription factors and downstream genes. Differential expression of H_2_O_2_-producing genes (*sod*) and H_2_O_2_-scavenging genes (*cat*) resulted in different H_2_O_2_ concentrations in SII and SIO. Some target genes of MSN2 (group A) showed upregulated expression in the sclerotium (inside), while others (group B) were up-regulated in hyphae (outside). Finally, Different morphogenesis (sclerotium formation in SII and hyphae growth in SIO) occurred. Genes marked with red indicated upregulation and of log2 (Fold Change) value was followed. Red and green arrows indicate upregulation or downregulation of expression, respectively. Black dotted arrows indicated unknown process. Ellipsis indicated unshown genes.

Transcriptome data analysis showed differential gene expression levels and metabolic characteristics between sclerotial and hyphal cells, which revealed the morphogenesis caused by different ROS stress at sclerotial initiation stage in *M. importuna*, as shown in Fig. 8. Gene expression related to H_2_O_2_-production was higher in the hyphae area (outside), while the expression of H_2_O_2_-scavenging genes was higher in sclerotial cells (inside), leading to different H_2_O_2_ concentrations in SII and SIO. Receptors in the cell membrane could receive the ROS signal, transmit it to kinases (MAPK pathway), which control transcription factor expression and their downstream genes. Expression levels of some MSN2 target genes (group A) were higher in the sclerotium (inside) than hyphae (outside), while other target genes (group B) showed opposite expression. Different morphogenesis occurred in SII and SIO: sclerotium formation in SII and hyphae growth in SIO. It has been reported that ROS concentration and response can affect root differentiation in *Arabidopsis*^51^. ROS play an important role in morphological development of sclerotia of fungi^11^. Therefore, it is of great significance to study how ROS regulate sclerotia formation in *Morchella*. Future studies entail the analysis key genes and metabolic pathways functions through genetic transformation to further reveal the molecular mechanism of ROS regulating sclerotia formation in *Morchella*.

## Materials and methods

### Fungal strain, culture and morphological observation

*M. importuna* strain CGMCC 5.2196 used in this work was routinely grown at 20 °C in potato dextrose agar (PDA) medium (200 g potatoes boiled in 1000 mL distilled water, then 20 g dextrose and 18 g agar were added to 1000 mL of potato extract water) in the dark. The internal transcribed spacer (ITS1-5.8S-ITS2 of nuclear ribosomal DNA) sequence was deposited in GenBank under accession number MH005092. Morphology observations were conducted with a dissecting microscope (SMZ1500, Nikon, Minato-ku, Tokyo, Japan). Microscopic preparations were made in clear lactic acid and observed with a Nikon Eclipse 80i microscope using differential interference contrast illumination (Nikon, Minato-ku, Tokyo, Japan).

### RNA extraction and library preparation

*M. importuna* strain CGMCC 5.2196 was incubated on PDA plates at 20 °C for 4 days in the dark. Fresh mycelia were collected for RNA isolation as described in our previous report^9^. Sclerotial and hyphal cells were mechanically broken using a Microsmash disrupter (Tomy Medico, Nerima-ku, Tokyo, Japan) at 4 °C, by 4 rounds of 40-s bead-beating at 5500 rpm. Total RNA was extracted using TRIzol reagent (Invitrogen, Carlsbad, CA, USA). Extracted RNA was then treated with RQ1 RNase-Free DNase (Promega, Madison, WI, USA).

A NanoPhotometer spectrophotometer (Implen, Baxter Avenue, Essex, UK) and an RNA Nano 6000 Assay Kit for the Agilent Bioanalyzer 2100 system (Agilent Technologies, Santa Clara, CA, USA) were used to evaluate RNA quality and concentration. Four libraries were generated using a NEB Next Ultra RNA Library Prep Kit (NEB, Ipswich, MA, USA) following the manufacturer’s recommendations.

### Transcriptome sequencing and data analysis

Library quality was assessed using an Agilent Bioanalyzer 2100 system. Paired-end 150 bp reads were sequenced using an Illumina HiSeqX-ten platform (Illumina Inc., San Diego, CA, USA) by Allwegene Technology Co., Ltd. (Haidian, Beijing, China).

Clean data was obtained by removing reads containing adapters and poly-Ns, and low-quality reads from the raw data. Clean reads were mapped to the *M. importuna* genome^10^ using TopHat v2.1.0^52^. Differential expression analysis was performed using DEGSeq R package^53^. A Q value < 0.01 and |log2 (Fold Change) |≥ 1 were set as the threshold for significantly differential expression. A heat map was generated using TBtools software (https://github.com/CJ-Chen/TBtools/releases)^54^.

Gene Ontology (GO) enrichment analysis of differentially expressed genes (DEGs) was performed using the GOseq R package^55^. KOBAS 3.0 was used to test statistical enrichment of DEGs in the Kyoto Encyclopedia of Genes and Genomes (KEGG) pathways^56,57^. Raw Illumina sequencing data was deposited in NCBI under bioproject GSE153704.

### Reverse-transcription (RT)-PCR and quantitative real-time PCR

RNA isolation and RT-PCR were performed as previously reported^9^. Gene sequences were searched from the JGI (https://genome.jgi.doe.gov/Morco1/Morco1.home.html). Oligonucleotide primers used are listed in Supplemental Table S1, and primers for *gapdh* were performed as previously reported^9^. Gel images were converted to 8-bit images, and the intensity of each band was measured using ImageJ software (http://rsb.info.nih.gov/ij). The intensity ratio of *hsp* gene expression to *gapdh* expression was calculated.

Expression levels of some genes were verified by qRT-PCR. cDNA was synthesized using HiScript III RT SuperMix (Vazyme Biotech, Nanjing, China). qRT-PCR was conducted using a CFX Connect Real-Time PCR System (Bio-Rad, Richmond, CA, USA). The 25 μL qPCR solutions contained 5 ng of cDNA, 0.1 μM primers, and 12.5 μL of SYBR qPCR Master Mix (Vazyme Biotech, Nanjing, China). *Actin* was used as an internal standard^58^. Relative gene expression levels were calculated using the 2^−ΔΔCT^ method^59^. Obtained data represented three biological replicates, with two technical replicates each.

### Ethical approval

This article does not contain any studies with human participants or animals performed by any of the authors.

## Supplementary Information

Supplementary Figures.

Supplementary Tables.
