# Supplementary material for: Comparative transcriptome analysis of cells from different areas reveals ROS responsive mechanism at sclerotial initiation stage in Morchella importuna
Source: Sci Rep. 2021 May 3;11:9418. doi: 10.1038/s41598-021-87784-w (PMC8093252; doi:10.1038/s41598-021-87784-w)
Supplement: Supplementary file 1 — Supplementary Figures. [file 41598_2021_87784_MOESM1_ESM.docx]

**Scientific Reports**

**Title of the article:**

**Comparative transcriptome analysis of cells from different areas reveals ROS responsive mechanism at sclerotial initiation stage in Morchella importuna**

Qizheng Liu^1^, Guoqiang He^2^, Jinkang Wei^2^, Caihong Dong^1^*

^1^State Key Laboratory of Mycology, Institute of Microbiology, Chinese Academy of Sciences, Beijing 100101, China

^2^Beijing Agricultural Technology Extension Station, Beijing 100029, China

* Corresponding author

Address: State Key Laboratory of Mycology

Institute of Microbiology, Chinese Academy of Sciences

NO.3 1st Beichen West Road, Chaoyang District, Beijing, 100101

China

E-mail: dongch@im.ac.cn

Telephone: +86-10-64806138

Fax: +86-10-64806138

OCRID: Dong Caihong 0000-0002-2558-3404

**Fig. S1**


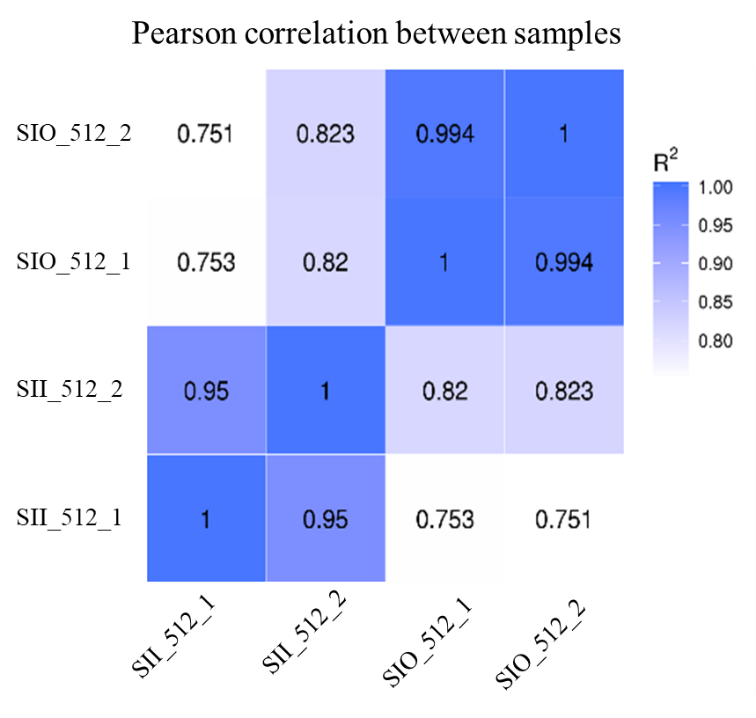


**Fig. S1** Pearson correlation analysis of sequencing data

The Pearson correlation coefficients were presented in each matrix.

**Fig. S2**


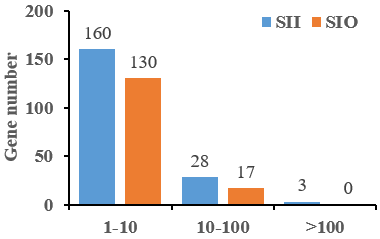


**Fig. S2** The number of unique genes with different expression levels

The X axis indicated three groups with different FPKM values (1-10,10-100 and more than 100). The numbers above the column were the number of unique genes.

**Fig. S3**


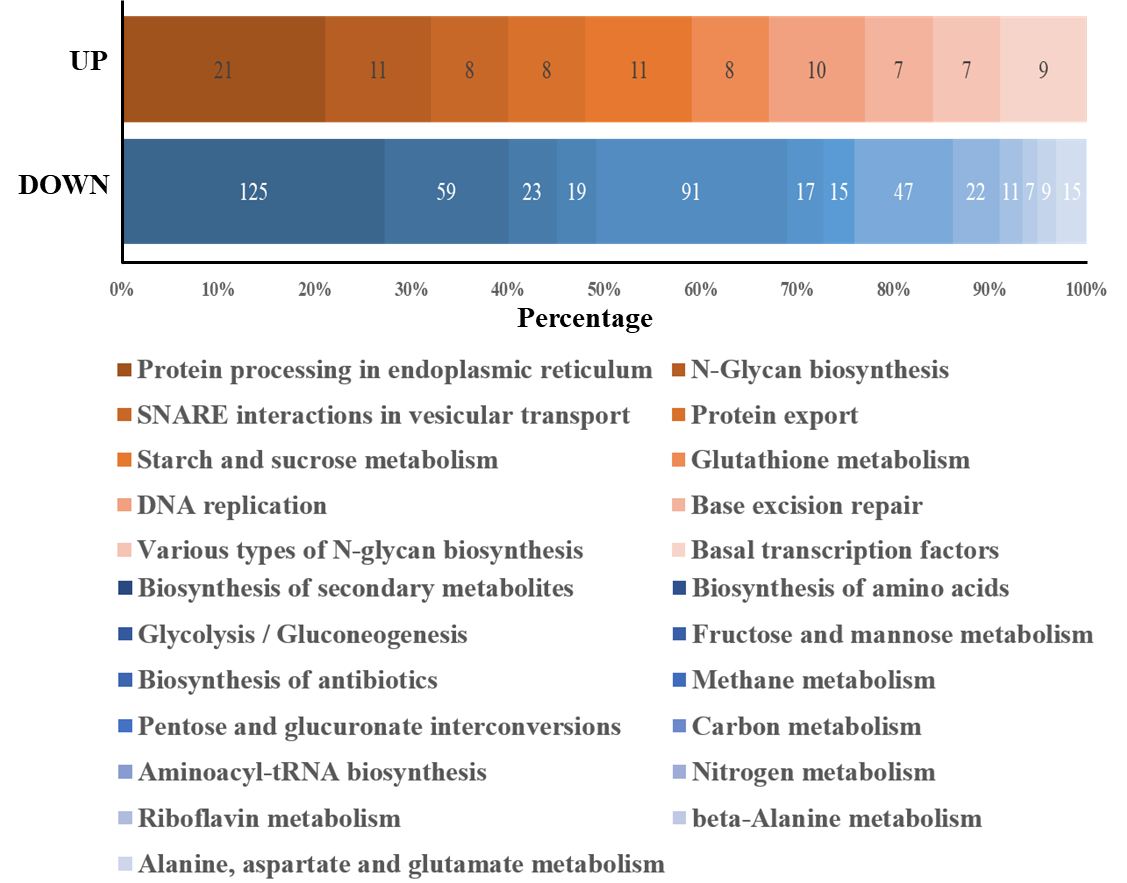


Fig. S3 The enriched KEGG pathway of DEGs compared between SIIO and SIO

‘Up’ indicated the enriched KEGG pathways of up-regulated DEGs and ‘down’ indicated enriched KEGG pathways of down-regulated DEGs. The number of genes in each KEGG pathway was indicated in the histogram. The different colors indicated the different KEGG pathways as the annotation.

**Fig. S4**


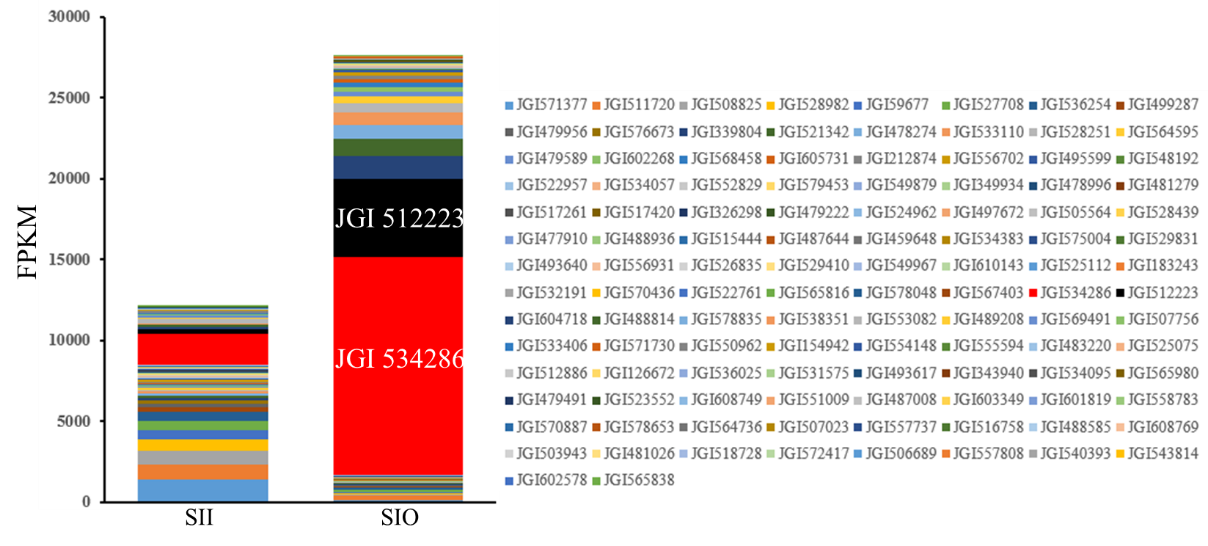


Fig. S4 FPKM values of DEGs in CAZy family

The histogram was obtained by accumulating the FPKM values of DEGs in CAZy family. Different colors represented different genes as annotation.

**Fig. S5**


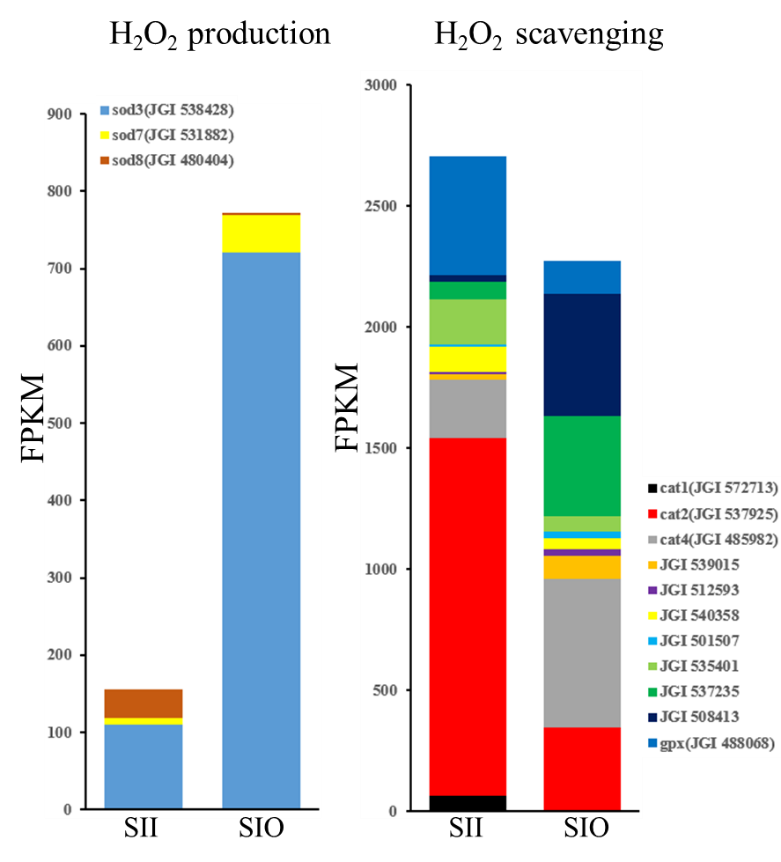


Fig. S5 FPKM value of DEGs related to H_2_O_2_ metabolism

The histogram was obtained by accumulating the FPKM values of DEGs related to H_2_O_2_- producing and H_2_O_2_-scvenging. Different colors represented different genes as annotation.

**Fig. S6**

**a**


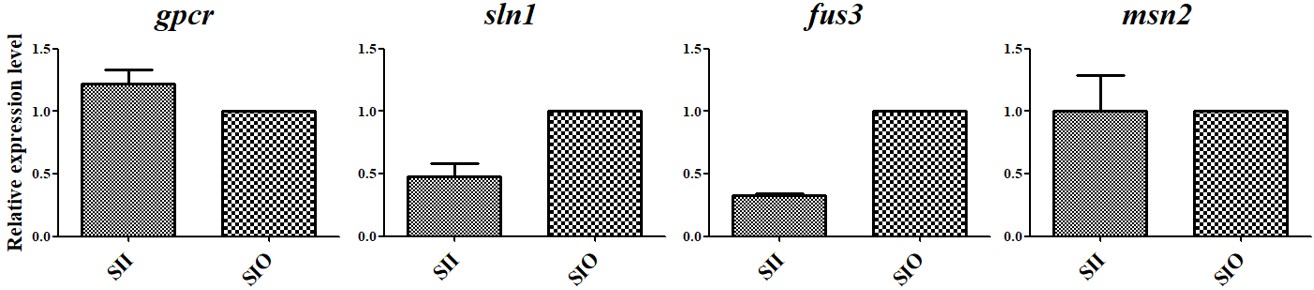


**b**


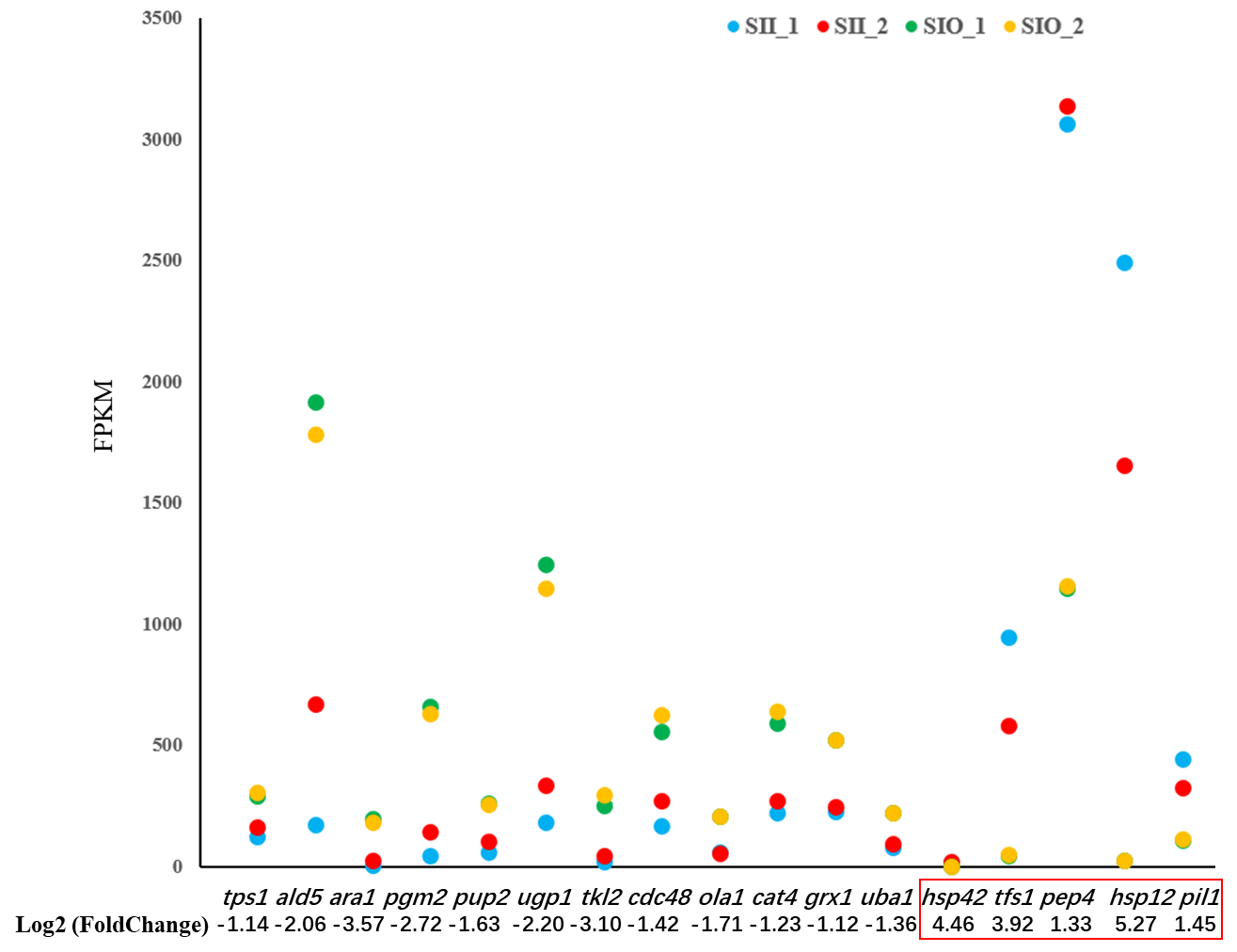


Fig. S6 Expression level of genes related to H_2_O_2_ -induced signal pathway

a. Relative expression level of *gpcr*, *sln1*, *fus3* and *msn2* in the samples of SII and SIO revealed by qRT-PCR

b. FPKM value of DEGs among the MSN2 target genes

The FPKM values of four samples were shown as dot with different color. The up-regulated genes in the sclerotia (SII) were indicated with red frame. The value of Log2 (Fold Change) were under the gene name.

**Fig. S7**


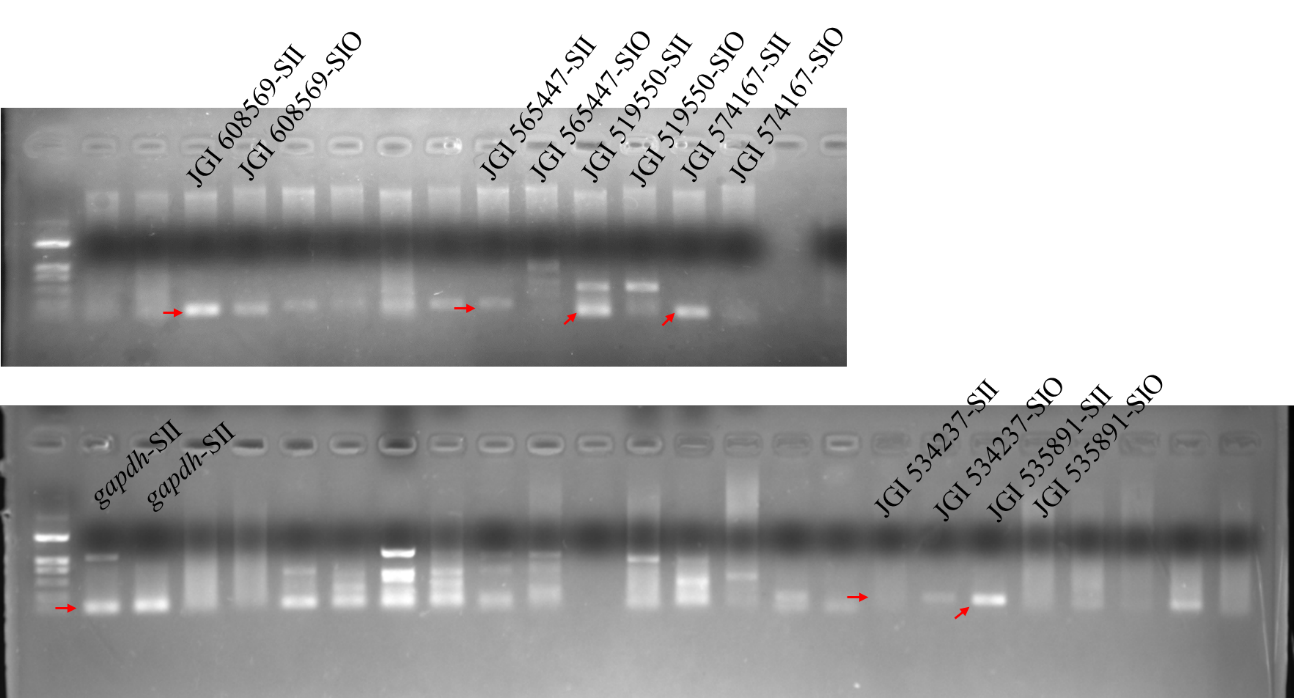


Fig. S7 RT-PCR electrophoresis graphs of *hsp* genes in SII and SIO samples

The red arrow points to the PCR products. Marked lanes by gene name are used in the data.
